# Free-standing TiO_2_ nanotubes decorated with spherical nickel nanoparticles as a cost-efficient electrocatalyst for oxygen evolution reaction

**DOI:** 10.1039/d0ra07563a

**Published:** 2020-12-23

**Authors:** Łukasz Haryński, Katarzyna Grochowska, Jakub Karczewski, Jacek Ryl, Jakub Rysz, Katarzyna Siuzdak

**Affiliations:** Centre for Plasma and Laser Engineering, The Szewalski Institute of Fluid-Flow Machinery Polish Academy of Sciences Fiszera 14 St. 80-231 Gdansk Poland lharynski@imp.gda.pl; Faculty of Applied Physics and Mathematics, Gdansk University of Technology Narutowicza 11/12 St. 80-233 Gdansk Poland; Faculty of Chemistry, Gdansk University of Technology Narutowicza 11/12 St. 80-233 Gdansk Poland; M. Smoluchowski Institute of Physics, Jagiellonian University Lojasiewicza 11 St. 30-348 Krakow Poland

## Abstract

Here, we report significant activity towards the oxygen evolution reaction (OER) of spherical nickel nanoparticles (NPs) electrodeposited onto free-standing TiO_2_ nanotubes (TNT) *via* cyclic voltammetry. It has been shown that simple manipulation of processing parameters, including scan rate and number of cycles, allows for formation of the NPs in various diameters and amounts. The polarization data with respect to transmission electron microscopy (TEM) allowed for determination of the diameter and propagation depth of the Ni NPs leading to the highest activity towards the OER with an overpotential of 540 mV at +10 mA cm^−2^ and Tafel slope of 52 mV per decade. X-ray photoelectron spectroscopy (XPS) indicates the presence of structure defects within Ni NPs whereas Mott–Schottky analysis provides information on the anodically shifted flat band potential and highly increased donor density. The obtained results along with literature studies allowed a proposal of the origin of the enhancement towards the OER. We believe that combination of transition metal-based NPs and TNT provides valuable insight on efficient and low-cost electrocatalysts.

## Abstract 2

In this work, we show the electrocatalytic activity towards the oxygen evolution reaction (OER) of spherical nickel nanoparticles (NPs) electrodeposited onto free-standing TiO_2_ nanotubes (TNT) *via* cyclic voltammetry.

## Graphical abstract

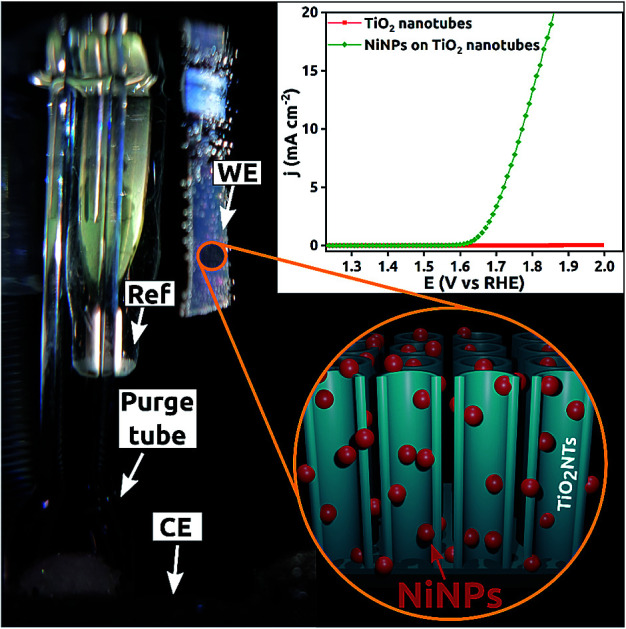

## Introduction

Nowadays, climate changes caused by the greenhouse effect and the decreasing number of non-renewable energy sources such as fossil fuels force the market to seek carbon-free fuels produced from renewables. This problem could be solved by the use of hydrogen gas directly generated from solar energy through water electrolysis.^1^ Its combustion provides high energy density, emitting at the same time steam vapor instead of carbon dioxide. Thus, it is a very promising and safe energy carrier for the environment. However, the process covers two half–cell reactions: HER (hydrogen evolution reaction) at a cathode and OER at an anode. Among them, the OER mechanism is more complex compared to the HER. Considering a metal oxide catalyst in alkaline solution,^2^ it can be described as a series of radical reactions in which hydroxide radicals are formed by one electron oxidation of hydroxyl anion on an active site (simply written as M), leading to the formation of adsorbed hydroxide:

1 ^−^OH → ˙OH + 1e^−^,

2 M + ˙OH → M˙OH,

3 (in total: M + ^−^OH → M˙OH + 1e^−^).

Once the hydroxide is formed on an active site, another hydroxyl radical lead to formation of oxide.

4 ^−^OH → ˙OH + 1e^−^,

5 M˙OH + ˙OH → M˙O˙ + H–O–H,

6 (in total: M˙OH + ^−^OH → M˙O˙ + H–O–H + 1e^−^).

Next, there are two pathways. In one pathway two oxides give oxygen molecule and reconstruct two active sites:

7 M˙O˙ + M˙O˙ → 2M + O=O.

In second pathway, hydroxyl radical reacts with the oxide giving hydroperoxide intermediate:

8 ^−^OH → ˙OH + 1e^−^,

9 M˙O˙ + ˙OH → M˙O–OH,

10 (in total: M˙O˙ + ^−^OH → M˙O–OH + 1e^−^).

Subsequently, another hydroxyl radical couples proton and electron from hydroperoxide giving water molecule and leading to reconstruction of the active site and oxygen evolution:

11 ^−^OH → ˙OH + 1e^−^,

12 M˙O–OH + ˙OH → H–O–H + M + O=O,

13 (in total: M˙O–OH + ^−^OH → H–O–H + M + O=O + 1e^−^).

Therefore, a complex mechanism involving 4 electrons is responsible for high overpotential of OER comparing to HER.

It is well-known that active surface area plays a significant role in catalysis. Therefore, the catalyst in a form of nanoparticles immobilized onto a substrate with large surface area seems to be reasonable approach. According to the literature,^3^ nickel species exhibit considerable activity towards OER. Moreover, nickel as an earth abundant transition metal representative, is available and low-cost. In turn, well-ordered TNT, considered as a substrate platform offer chemical stability,^4^ high surface area,^5^ directional charge transfer^6^ and the possibility of precise control of the geometric features of the tubular structure during electrochemical synthesis,^7^ commonly known as anodization. Another advantage of anodized TNT is that the NTs are directly grown onto the conductive substrate (titanium foil). Thanks to that, no further detach and transfer are needed. To the best of our knowledge, TNT decorated with spherical nickel NPs have not been thoroughly electrochemically investigated as a candidate for OER electrocatalyst.

Nickel NPs can be produced *via* hydrothermal synthesis^8–13^ and afterwards transferred onto the NTs or directly electrochemically deposited.^14–18^ The electrochemical approach can be realized *via* constant voltage deposition,^14^ pulsed current electroplating^15^ or cyclic voltammetry (CV).^16^ According to Nasirpouri *et al.*,^18^ DC electrodeposition results in NPs formation mainly on the top of the nanotubes whereas CV leads to complete coverage of the NTs. Among the described methods, CV offers precise control of size and amount of NPs through the manipulation of potential range, scan rate and number of cycles.

In this work, we report the formation of nickel oxide/hydroxide spherical NPs onto free standing TiO_2_ nanotubes (Ni-TNT) *via* CV method preceded by electrochemical hydrogenation in order to improve samples conductivity. The electrode fabricated under optimized processing parameters, including scan rate and number of cycles, exhibits considerable activity towards OER with overpotential of 540 mV at + 10 mA cm^−2^ and Tafel slope of 52 mV per decade. Morphology, crystallinity, and chemical nature of the obtained electrodes were revealed by scanning electron microscopy (SEM), X-ray diffraction (XRD) and XPS, respectively. Nickel NPs penetration depth was discussed according to TEM and secondary ion mass spectrometry (SIMS) profiles. The Mott–Schottky analysis indicates significant increment in donor density accompanied by positive shift of flat band potential of Ni-TNT comparing to TNT which is recognized to be a key factor responsible for OER activity.

## Experimental

Well-ordered free-standing TNT were obtained *via* one-step anodization in glycol diethylene electrolyte with 0.3 wt% NH_4_F and 0.5 wt% HF. Prior to the process, Ti foil (Strem. 99.7%) was cut into (3.5 × 2 cm^2^) samples. Afterwards, they were ultrasonically cleaned in sequence in three solvents: acetone, ethanol and water for 10 minutes in each one. Then, samples were rinsed with isopropanol and dried in a stream of air. The anodization was realized in a two-electrode setup, where the Ti foil served as an anode and a platinum mesh as a cathode. During the process, 20 V was maintained for 5 h. The temperature was set to 40 °C and was controlled by the thermostat (Julabo F-12) working in the flow-mode. The distance of 2 cm between electrodes was kept. After the anodization, pristine TNT were rinsed with ethanol, immersed in ethanol for 30 minutes and allowed to dry in the air. Next, samples were placed in a tubular furnace where they were thermally annealed to 450 °C with a heating rate of 2 °C min^−1^. Once the temperature was reached it was maintained for 2 h. After that time samples were allowed to cool down freely to the ambient temperature.

Calcined TNT were hydrogenated electrochemically in 0.5 M Na_2_SO_4_ at ambient temperature. The process was carried out in a three-electrode setup where platinum mesh served as a counter electrode, TNT sample as a working electrode and Ag/AgCl/0.1 M KCl as a reference electrode. Hydrogenation was realized using the chronoamperometry technique at −3.0 V *vs.* Ag/AgCl/0.1 M KCl for 3 minutes. After the process, samples were rinsed with deionized water and dried in a stream of cold air.

Nickel electrodeposition was performed *via* the CV based procedure in an aqueous solution containing 0.05 M NiSO_4_ and 0.32 M H_3_BO_3_ at ambient temperature. The electrodes arrangement was the same as in the case of hydrogenation. The CVs were performed in the potential range from +0.05 V to −1.2 V in a forward and return sweeps. The scan rates were set to 60, 90, 120 mV s^−1^ and for each scan rate, 1 and 3 scans were registered. According to those parameters, the samples were labeled as (*X*–*Y*)Ni-TNT, where X stands for the scan rate whereas Y for the number of scans. Sample labels corresponding to particular CV electrodeposition parameters are shown in Table 1. Afterwards, samples were rinsed with deionized water and dried in a stream of cold air.

**Table 1:** Sample labels corresponding to particular electrodeposition parameters

CV electrodeposition parameters	Sample
—	TNT
60 mV s^−1^, 1 scan	(60–1)Ni-TNT
60 mV s^−1^, 3 scans	(60–3)Ni-TNT
90 mV s^−1^, 1 scan	(90–1)Ni-TNT
90 mV s^−1^, 3 scans	(90–3)Ni-TNT
120 mV s^−1^, 1 scan	(120–1)Ni-TNT
120 mV s^−1^, 3 scans	(120–3)Ni-TNT

SEM images of the samples in top view and cross-section were captured utilizing the Schottky field emission scanning electron microscopy (FEI Quanta FEG 250) with an ET secondary electron detector. The acceleration voltage was kept at 10 kV.

TEM images were obtained using (JEOL ARM-200F, Japan) equipped with an energy-dispersive X-ray (EDX) spectroscopy detector. Sample preparation was performed by mechanically scratching the surface of the samples using a flat spatula directly on the copper grid. Scratching was used in order to avoid the detachment of the NPs by sonication. Additionally, in order to discard possible melting and increment of size of the NPs, the examination was carried out in low accelerating voltage (80 kV).

The phase composition of the samples was analyzed by X-ray diffractometer (Bruker D2 Phaser 2^nd^ generation) using CuK_α_ radiation and a LynxEye XE-T detector at room temperature.

In order to reveal the propagation depth of the chemical composition within the material profile, the Time of Flight Secondary Ion Mass Spectrometer (TOF SIMS: ION-TOF GmbH) working in dual beam mode was employed. As the analysis and sputter beams, the Bi^+^ 30 keV and Cs^+^ 2 keV ions were utilized, respectively, both incident at 45° to the surface normal. The Cs^+^ supper beam was rastered over (350 × 350 μm^2^) while the (100 × 100 μm^2^) central region of the sputter crater was analyzed with the Bi^+^ ions. Since the primary Bi ions result in anions formation, the negatively charged secondary ions were collected using time of flight mass spectrometer. SurfaceLab 6 software (ION-TOF GmbH) was used to analyze the change of intensity of characteristics signals during the sputtering. The following signals were selected for verification of the uniformity and chemical composition from the top of the NTs to the titanium substrate: Ni^−^ (*m*/*z* = 57.93), ^46^TiO^−^ (*m*/*z* = 61.94) and ^18^O^−^ (*m*/*z* = 17.99).

The chemical composition of modified material was inspected using the X-ray photoelectron spectroscope (Escalab 250Xi, ThermoFisher Scientific). The device was equipped with a monochromatic AlKα source with charge neutralization implemented by means of a flood gun. Calibration was based on the peak characteristic for neutral carbon (*E*_bin_ = 284.6 eV). The high-resolution spectra of Ti 2p, O 1s, Ni 2p, C 1s for the (60–3), (90–3) and (120–3)Ni-TNT were measured. Please note that Ni NPs label is not related to the actual chemical nature of the nanoparticles which is described later on in the manuscript.

The electrochemical measurements were carried out utilizing Autolab PGStat 302 N potentiostat–galvanostat system (Metrohm Autolab). A three-electrode system was chosen where the Ni-TNT and the TNT served as a working electrode, Ag/AgCl/0.1 M KCl as a reference electrode and platinum mesh as a counter electrode. Samples were examined in 0.5 M NaOH. Prior to the tests, the solution was deaerated with argon 5.0. During experiments, a continuous flow of Ar above the solution was kept. The recorded potential values (*iR* drop not corrected) were subsequently normalized to the reference hydrogen electrode (RHE) according to the following equation:^19^

14 *E*_RHE_ = *E*_Ag/AgCl/0.1 M KCl_ + *E*_Ag/AgCl/0.1 M KCl_^0^ + 0.059pH

where *E*_Ag/AgCl/0.1 M KCl_^0^ = 0.288 V and pH = 13.61 for 0.5 M NaOH solution. The pH value was estimated using pH meter MP-103. The overpotentials were calculated as *E*_RHE_ − 1.229 V.

CV measurements were performed in the potential range from −0.8 to 0.8 V with the scan rate of 50 mV s^−1^. Linear voltammetry (LV) curves were registered from −1.0 to +1.0 V with the scan rate of 5 mV s^−1^. Chronopotentiometry data was collected at +10 mA cm^−2^ for 12 h.

The electrochemical impedance spectroscopy (EIS) measurements employed for the Mott–Schottky analysis were carried out for the (90–3)Ni-TNT and the TNT. Prior the measurements, the investigated samples were not subjected to any preliminary treatment or measurement and their potential was held to reach a steady-state conditions. The EIS data was recorded for the single frequency of 1000 Hz in the potential range from +0.2 to −1.0 V *vs.* Ag/AgCl/0.1 M KCl using a 10 mV amplitude of the AC signal. The capacitance of the space charge layer was further calculated from the imaginary part of the measured impedance using the following equation:15
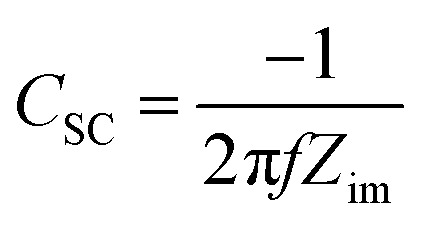
where *f* stands for the frequency of the AC signal and *Z*_im_ for the imaginary part of impedance. The donor density (*N*_d_) was calculated according to the Mott–Schottky space charge capacitance of the semiconductor theory given by:16
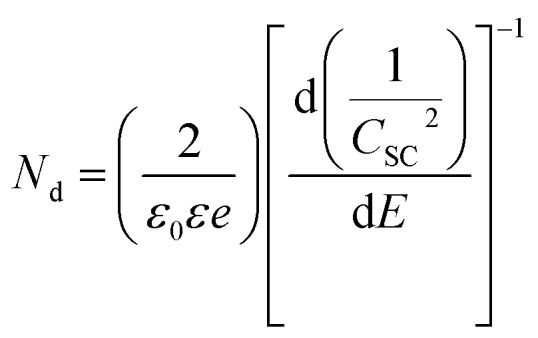
where *ε*_0_ is vacuum permittivity, *ε* is the dielectric constant of TiO_2_, *e* is electron charge and *E* is the applied potential; in calculations *ε*_0_ = 9.85 × 10^14^ F cm^−1^, *ε* = 38,^20^*e* = 1.602 × 10^−19^ C were used.

## Results and discussion

**Fig. 1:**
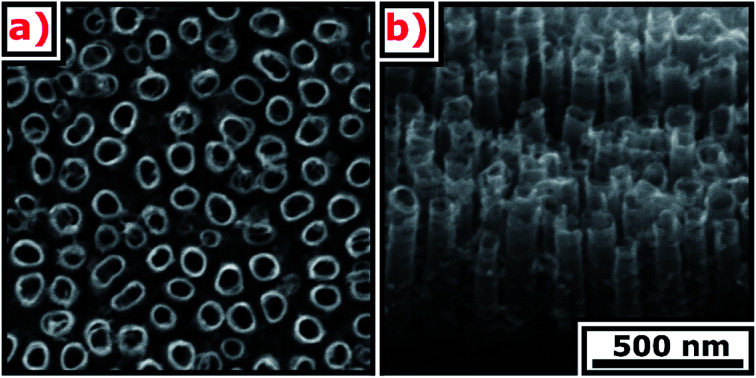
SEM images of the TNT (a) top view, (b) cross-section.

SEM images of the TNT are presented in Fig. 1. The ordered layer is composed of spaced nanotubes with 57 ± 9 nm internal diameter, 12 ± 3 nm wall thickness, 320 ± 4 nm length, and 42 ± 15 nm distance between external walls.

**Fig. 2:**
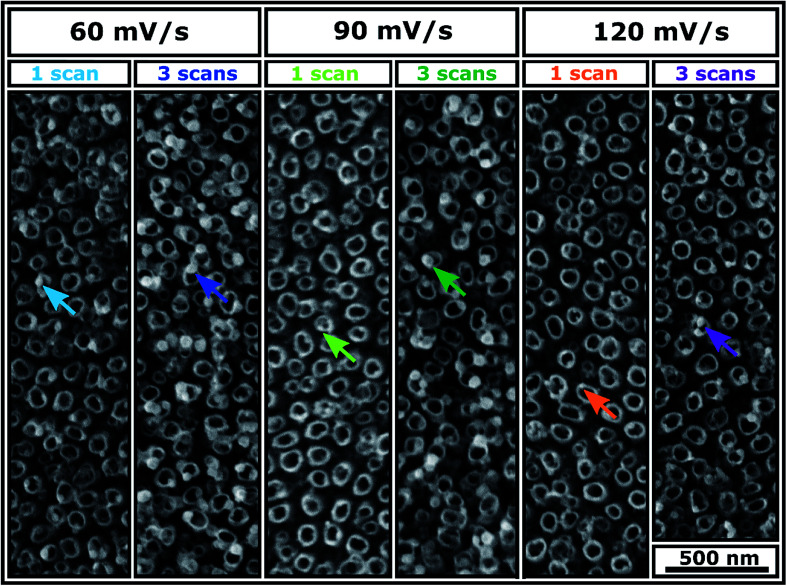
SEM images of Ni-TNT obtained from different electrodeposition parameters.

SEM images of Ni-TNT are shown in Fig. 2. Since electrodeposition does not affect the morphology of the NTs, the geometric features of the tubular architecture are no longer considered. It can be clearly seen that the electrochemically supported synthesis route resulted in the formation of spherical nanoparticles. Their chemical composition and penetration depth are described in sections dedicated to the analysis of XPS spectra and TEM supported by EDX mapping along with SIMS profile, respectively. The impact of the processing parameters, *i.e.* scan rate and number of cycles on the Ni NPs formation is as follows: (i) increase in scan rate facilitates formation of smaller NPs and does not impact their number significantly (ii) multiplication of the number of cycles for a certain scan rate contributes to the formation of more NPs with a larger size. Thus, one can conclude that the NPs nuclei aggregate into clusters with the subsequent cycles. The aggregative growth during electrodeposition has already been reported in the literature.^21^ However, this is contradictory to the observation reported by Nasirpouri *et al.*^18^ Instead of the aggregative growth they reported increase in the number of NPs with the same diameter along with subsequent cycles.

**Fig. 3:**
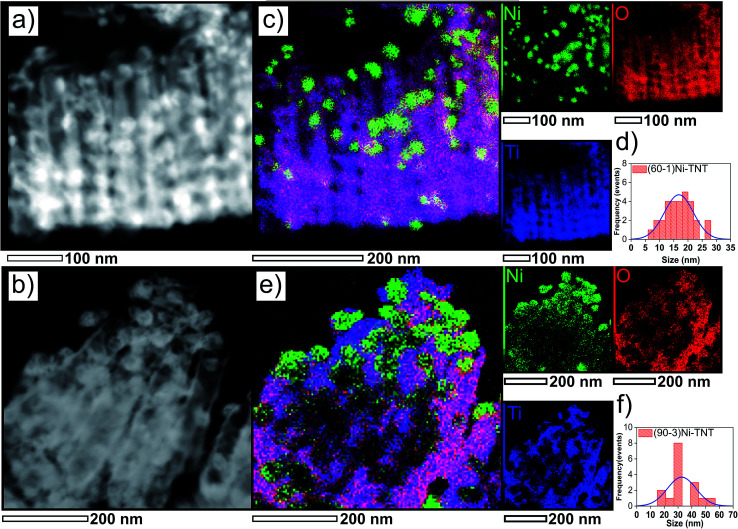
TEM images analysis of the (60–1)Ni-TNT (a, c and d) and (90–3)Ni-TNT (b, e and f); here: (a and b) represents dark field images, (c and e) EDX elemental mapping images and (d and f) histograms displaying estimated Ni NPs size.

High-resolution TEM images of two samples, the (60–1) and (90–3)Ni-TNT, exhibiting the highest activity towards OER described later on are shown in Fig. 3a and b, respectively. The simultaneous EDX mapping of the (60–1)Ni-TNT (Fig. 3c) clearly demonstrates formation of NPs with an average size of 17 ± 5 nm (Fig. 3d) alongside the NTs. Their distribution is not uniform and is mainly limited to half the length of the NTs from the top of the NTs. In case of the (90–3)Ni-TNT (Fig. 3e), larger Ni NPs of 34 ± 10 nm (Fig. 3f) are formed. On the contrary to the (60–1)Ni-TNT, agglomeration of Ni NPs is visible on the top of the NTs. Formation of large size agglomerates could be responsible for preventing their further penetration along the NTs.

**Fig. 4:**
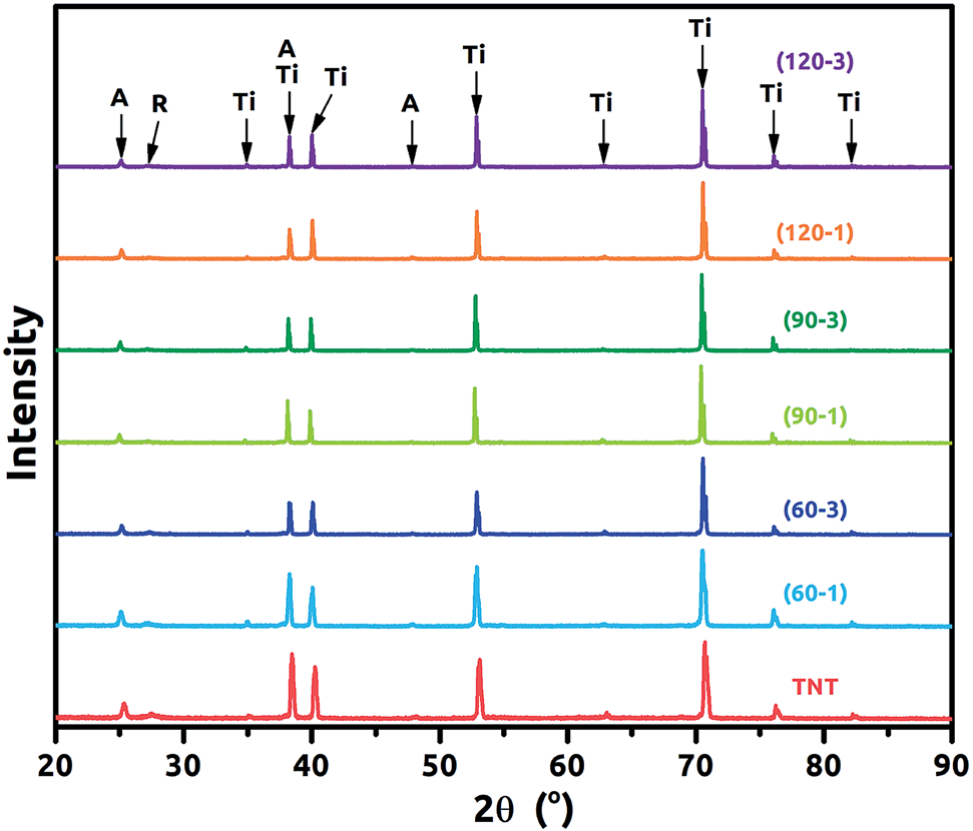
XRD patterns for the TNT and Ni-TNT (A – anatase, R – Rutile, Ti – titanium).

XRD patterns of the TNT and Ni-TNT are presented in Fig. 4. For all samples diffraction peaks corresponding to anatase, rutile and titanium can be observed and they are listed in Table 2. Particular peak positions were identified according to the [ICDD PDF-4+] and marked with black arrows. Anatase to rutile phase ratio were calculated according the A. Spurr and H. Myers formula^22^ and it is 65 to 35%. In general, the diffraction peaks intensity of anatase and rutile are much lower in comparison to those originating from titanium used here as a substrate. Taking into account that the oxide layer is composed of *ca.* 320 nm tubular layer grown on 0.127 mm titanium foil, such proportions seem to be reasonable. It is worth noting that, no signal corresponding to Ni or NiO was registered for Ni-TNT as the amount of Ni NPs is relatively small comparing to the whole volume of the sample.

**Table 2:** The identified position of peaks corresponding to anatase, rutile phases and titanium substrate

Phase	Anatase	Rutile	Titanium
Angle (°)	25.1; 38.3; 47.8	27.3	35.0; 38.3; 40.0; 62.8; 70.5; 76.1; 82.2

**Fig. 5:**
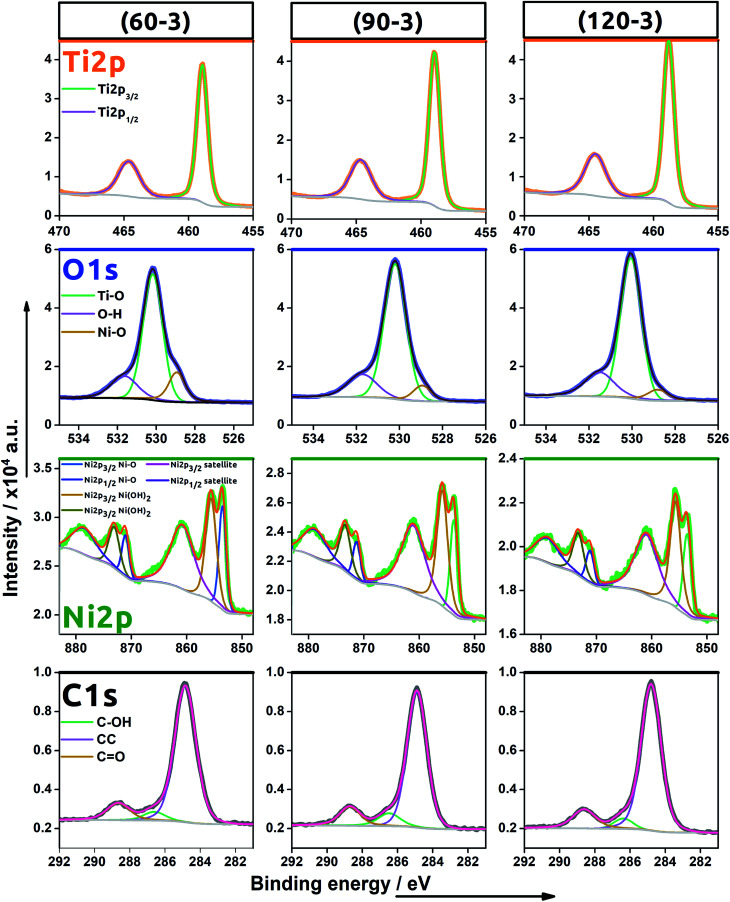
High resolution XPS spectra recorded for energy range for titanium, oxygen, nickel, carbon for (60–3), (90–3) and (120–3)Ni-TNT.

XPS studies were carried out to confirm the successful TNT decoration with Ni NPs using the electrochemical protocol and to reveal the chemical nature of those species. Since the subsequent cycles increase only an amount of the nanoparticles which nuclei aggregate into clusters, the XPS spectra were recorded for samples prepared by performing 3 scans at different scan rates: 30, 90 and 120 mV s^−1^ (see Fig. 5). Here, signals typical for titanium, oxygen, nickel and carbon were found. The localisation of all the peaks is listed in Table 3.

**Table 3:** The location of the signals maxima found onto the XPS spectra for (60–3), (90–3) and (120–3)Ni-TNT within titanium, oxygen, nickel and carbon binding energy ranges

Element	Orbital	Binding energy (eV)		
		(60–3)Ni-TNT	(90–3)Ni-TNT	(120–3)Ni-TNT
Titanium	Ti 2p_1/2_	464.6	464.7	464.6
	Ti 2p_3/2_	458.9	458.9	458.8
Oxygen	O 1s	530.2; 531.7; 528.9	530.2; 531.7; 528.9	530.0; 531.5; 528.8
Nickel	Ni 2p_1/2_	871.1; 873.2; 878.8	871.2; 873.4; 879.0	871.2; 873.3; 879.1
	Ni 2p_3/2_	853.5; 855.6; 860.9	853.7; 855.8; 861.1	853.6; 855.7; 861.2
Carbon	C 1s	284.9; 286.6; 288.7	284.9; 286.5; 288.7	284.8; 286.4; 288.6

As indicated in the spectra recorded within the binding energy range typical for Ti 2p, the signal was fitted by the one doublet of Ti 2p_1/2_ and Ti 2p_3/2_ peaks characteristic for the Ti–O bond in titanium dioxide.^23^ No signal typical for Ti^3+^ was found confirming that activation of the material *via* electrochemical hydrogenation did not lead to the permanent reduction of the Ti oxidation state. Regarding the oxygen spectra, the registered signal is composed of three bands with maxima found at 531.7 eV, 530.2 eV and 528.9 eV. The prominent one is attributed to the oxygen found in the titania lattice (Ti–O)^24^ while the peak located at the highest binding energy is assigned to the oxygen present in hydroxyl groups^25^ or some irreversibly adsorbed water molecules onto the material surface.^26^ The last signal arises due to the Ni–O bond present in nickel oxide as was also reported by Yu *et al.*^24^ and Guo *et al.*^26^.

In the binding energy range of 883–848 eV, one may find the clear signals typical for nickel 2p orbital that confirms successful deposition of nickel species into the TiO_2_NTs host matrix. The whole recorded Ni spectra were fitted by three doublets composed of Ni 2p_1/2_ and Ni 2p_3/2_ signals localized at (i) 853.6 eV and 871.2 eV, (ii) 855.7 eV and 873.3, (iii) 860.8 eV and 878.8 eV, respectively. The first doublet can be attributed to Ni^2+^ in the typical Ni–O octahedral bonding configuration while the second could be ascribed to the Ni^2+^ vacancy induced Ni^3+^ ion and/or interstitial oxygen in NiO^27^ or even interpreted as nickel hydroxide.^28^ It is worth mentioning that the presence of vacancies in metal oxides can enhance electrocatalytic water splitting since it reduces the free energy of reaction substrate adsorption.^29^ The peaks found for Ni 2p_1/2_ and Ni 2p_3/2_ orbitals at the highest binding energy, namely 878.8 eV and 860.9 eV are assigned to the satellite peaks characteristic for Ni^2+^.^30^

Finally, the spectra typical for the carbon species were also found. Since the anodization process has been realized in the organic/water electrolyte solution, some carbon residues remain despite the applied calcination.^31^ Moreover, the adventitious hydrocarbon from the XPS instrument itself should be also taken into consideration.^32^

Comparing the obtained spectra, no distinct difference between the (60–3), (90–3) and (120–3)Ni-TNT have been observed. Therefore, increase in scan rate does not affect the chemical nature of the electrodeposited nickel nanoparticles.

**Fig. 6:**
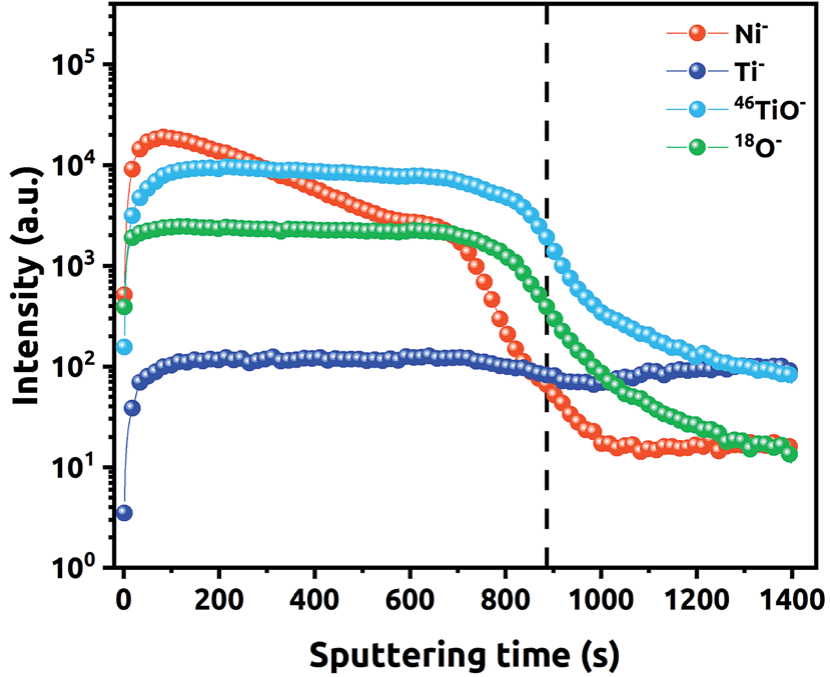
SIMS composition *vs.* depth profile of (90–3)Ni-TNT.

Penetration depth of Ni NPs along the NTs was also studied *via* SIMS. The recorded depth profiles for different ions detected during the investigation of the (90–3)Ni-TNT are shown in Fig. 6. Some initial variations, that can be observed, are related to the interaction of Cs^+^ ions with the outermost part of the sample. Particular signals intensity along with increasing sputtering time correspond to the penetration depth. Once the sputtering time reaches slightly less than 900 s, the significant drop in the ^46^TiO_2_^−^ signal intensity can be observed. This indicates that the height distribution of the NTs is very narrow and the titania/Ti interface has been reached. Considering the run of registered Ni^−^ ions, one can observe that the signal decreases gradually from the surface to 700 s of the sputtering period, and then, significant drop takes place. This changes are not in line with ^46^TiO^−^ and ^18^O^−^ signals. As it has been observed on TEM images, Ni NPs in the (90–3)Ni-TNT are located mainly on the top of the NTs. Therefore, because of the material heterogeneity (more Ni in the surface region), the constant sputtering time results in different etching rate of the material across the TNT layer.

**Fig. 7:**
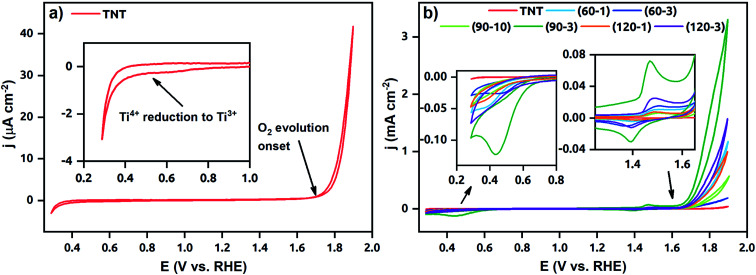
Cyclic voltammograms recorded for (a) the TNT and (b) both the TNT and Ni-TNT in 0.5 M NaOH.

Cyclic voltammograms of the TNT and Ni-TNT recorded in 0.5 M NaOH are presented in Fig. 7. Due to the low current density of the TNT, the voltammogram is being distinguished in Fig. 7a. Here, a typical run for TNT in alkaline solution, including peak onset at about +0.5 V *vs.* RHE towards the cathodic direction related to the Ti^4+^ reduction,^33^ very low capacitive current density in the range from *ca.* +0.5 to +1.6 V and sharp current density increment from *ca.* 1.6 V towards anodic direction originating from OER can be seen. Higher cathodic current densities in the range from about 0.3 V to 0.7 V were in turn registered for all Ni-TNT samples (see Fig. 7b). Additionally, a distinct peak located at +0.44 V can be seen for the (90–3)Ni-TNT sample. According to the literature,^16^ it is related to the Ni^2+^ to Ni^0^ reduction. Similarly to the TNT the low capacitive region from *ca.* +0.5 to 1.6 V is observed. Within that range, the reversible redox reactions at +1.47/+1.39 V related to the Ni(OH)_2_/NiOOH redox system are observed.^16^ Further current density increment observed from *ca.* +1.6 V towards anodic direction are related to the OER. The highest activity is exhibited by (90–3)Ni-TNT (*ca.* 3.3 mA cm^−2^ at +1.9 V). Here, the TNT shows almost negligible current density (*ca.* 42 μA cm^−2^ at 1.9 V). Considering cyclic voltammograms in 1 M KOH for NiO_*x*_ doped TNT presented in the literature,^27^ the current density reaches only 0.045 mA cm^−2^ at *ca.* +0.8 V *vs.* Ag/AgCl (about +1.9 V *vs.* RHE) which is significantly lower comparing to the value registered for the (90–3)Ni-TNT.

**Fig. 8:**
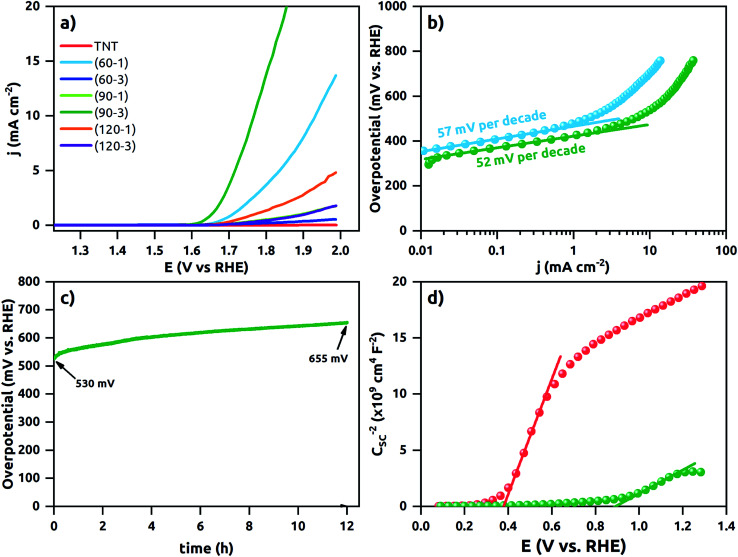
(a) Polarization data for the TNT and the Ni-TNT electrodes, (b) Tafel plots of the polarization curves of the (60–1)Ni-TNT and (90–3)Ni-TNT, (c) time dependence of overpotential under static current density of +10 mA cm^−2^ registered for the (90–3)Ni-TNT, (d) Mott–Schottky plots for the TNT and Ni-TNT. All electrochemical measurements were performed in deareated 0.5 M NaOH solution.

The polarization curves are shown in Fig. 8a. The TNT does not reach 10 mA cm^−2^ within the potential window up to +2.0 V *vs.* RHE. It demonstrates that it is electrocatalytically inactive for water oxidation kinetic. Similarly, (60–3), (90–1), (120–1) and (120–3)Ni-TNT samples are recognized as inactive and therefore they are not considered in further electrochemical investigations. In contrast, (60–1) and (90–3)Ni-TNT exhibit overpotentials of about 700 and 540 mV at +10 mA cm^−2^, respectively. This is 400 and 240 mV more than the RuO_2_ and IrO_2_ commercial OER catalysts exhibiting overpotential of +300 mV + 10 mA cm^−2^ in alkaline solution, respectively. As it can be observed on TEM images, the (90–3)Ni-TNT contains larger Ni NPs located mostly on the top of the NTs whereas in the case of the (60–1)Ni-TNT contains less Ni NPs are present alongside the NTs. Therefore, the localization of Ni NPs, particularly on the surface, could be a key factor responsible for the enhanced OER activity. Also, one can conclude that the reaction takes place at the interface between the top of the NTs and an electrolyte instead of on the sidewall surface of the NTs.

The linear portions of Tafel plots are presented in Fig. 8b. The data was fit to the Tafel equation yielding Tafel slopes of ∼57 and ∼52 mV per decade for (60–1) and (90–3)Ni-TNT, respectively. To investigate the stability during OER, the +10 mA cm^−2^ was maintained for 12 h and corresponding potential was measured. The (90–3)Ni-TNT sample was chosen for testing as it exhibits the highest OER activity with the lowest overpotential and Tafel slope. In Fig. 8c, time-dependent overpotential data is displayed. Here, the overpotential increases from *ca.* 530 to 655 mV at +10 mA cm^−2^ after 12 h. According to the Medway *et al.*,^34^ potential cycling across the nickel hydroxide/nickel oxyhydroxide redox pair results in NiO layer thickening which causes drop in the double layer capacitance. Considering that such redox pair has been observed for our samples (see right inset in Fig. 6b) and the potential during chronopotentiometry at +10 mA cm^−2^ exceeds the oxidation peak, the decrease in electrocatalytic activity is assigned to the voltage drop across the double layer. Mott–Schottky analysis was utilized in order to reveal the electronic properties of the most active sample towards OER comparing to the TNT. The impedance data was collected in 0.5 M NaOH solution within the range from +1.29 to 0.08 V *vs.* RHE, where according to the obtained cyclic voltammograms, no faradaic reaction has been observed. The Mott–Schottky plots are shown in Fig. 8d and flat band potential (*E*_fb_) is indicated in the place where the tangent to the plot intersects the potential axis. The calculated *N*_d_ are 3.5 ×10^20^ cm^−3^ and 7.3 ×10^19^ for the (90–3)Ni-TNT and the TNT, respectively. Thus, it can be assumed that nearly 5-fold increment originates from the Ni NPs. According to the literature,^35^ increase in electron density near NPs can promote catalytic activity since it reduces the free energy of substrate adsorption. Therefore, it is assumed that high concentration of *N*_d_ in the Ni NPs is responsible for the OER activity. Considering the *E*_fb_ of the (90–3)Ni-TNT, a positive shift of about +0.5 V in comparison to the TNT is observed. This anodic shift can be ascribed to the raise of Fermi level towards the conduction band^36^ caused by the presence of Ni NPs.

## Conclusions

Summarizing, in the present work a facile fabrication of electrocatalyst based on free-standing TNT decorated with spherical Ni NPs *via* subsequent electrochemical processes (anodization, hydrogenation and electrodeposition) has been demonstrated. SEM images show that during the electrodeposition size of Ni NPs depends not only on scan rate (as literature suggests) but also on number of cycles. XPS measurements established the chemical nature of the nickel NPs as oxidized ones. The obtained spectra suggest the presence of structure defect. Polarization data shows that the highest activity towards OER with overpotential of 540 mV at +10 mA cm^−2^ (which is 240 mV over RuO_2_ and IrO_2_ benchmarks of OER electrocatalyst in alkaline solution) and Tafel slope of 52 mV per decade is being reached for Ni NPs with a diameter of 28 ± 7 nm located mainly on the top of the NTs, as indicated by TEM inspection. The Mott–Schottky analysis reveals nearly 5-fold increased donor density and anodic shift of *E*_fb_ by about 0.5 V for the (90–3)Ni-TNT comparing to the TNT. It is assumed that high carrier concentration within Ni NPs along with structure defects could be responsible for significant OER activity. We believe that results of our work will contribute to wider applications of TNT as a platform for transition metal oxides based electrocatalyst.

## Conflicts of interest

The authors declare no competing financial interest.

## Supplementary Material
